# Physico-Chemical, Thermal, and Electrochemical Analysis of Solid Polymer Electrolyte from Vegetable Oil-Based Polyurethane

**DOI:** 10.3390/polym13010132

**Published:** 2020-12-30

**Authors:** Siti Rosnah Mustapa, Min Min Aung, Marwah Rayung

**Affiliations:** 1 Department of Chemistry, Faculty of Science, University Putra Malaysia, Serdang 43400, Selangor, Malaysia; sitivild89@gmail.com; 2 Unit of Chemistry, Centre of Foundation Studies for Agriculture Science, University Putra Malaysia, Serdang 43400, Selangor, Malaysia; 3 Institute of Forestry and Forest Products, University Putra Malaysia, Serdang 43400, Selangor, Malaysia; marwahrayung@yahoo.com

**Keywords:** jatropha oil, polyurethane, polyol, solid polymer electrolyte, electrochemical analysis

## Abstract

In this paper, we report the preparation of bio-based polyurethane (PU) from renewable vegetable oil. The PU was synthesized through the reaction between jatropha oil-based polyol and isocyanate in a one-shot method. Then, lithium perchlorate (LiClO_4_) salt was added to the polyurethane system to form an electrolyte film via a solution casting technique. The solid polymer electrolyte was characterized through several techniques such as nuclear magnetic resonance (NMR), Fourier transforms infrared (FTIR), electrochemical studies, thermal studies by differential scanning calorimetry (DSC), and dynamic mechanical analysis (DMA). The NMR analysis confirmed that the polyurethane was successfully synthesized and the intermolecular reaction had occurred in the electrolytes system. The FTIR results show the shifting of the carbonyl group (C=O), ether and ester group (C–O–C), and amine functional groups (N–H) in PU–LiClO_4_ electrolytes compared to the blank polyurethane, which suggests that interaction occurred between the oxygen and nitrogen atom and the Li+ ion as they acted as electron donors in the electrolytes system. DSC analysis shows a decreasing trend in glass transition temperature, *T_g_* and melting point, *T_m_* of the polymer electrolyte as the salt content increases. Further, DMA analysis shows similar behavior in terms of *T_g_*. The ionic conductivity increased with increasing salt content until the optimum value. The dielectric analysis reveals that the highest conducting electrolyte has the lowest relaxation time. The electrochemical behavior of the PU electrolytes is in line with the *T_g_* result from the thermal analysis.

## 1. Introduction

Polyurethane (PU) is one of the polyester/polyether polymers made by reacting polyols with the isocyanates group. PU possess good properties such as high mechanical strength, excellent abrasive resistance, toughness, low-temperature flexibility, chemical and corrosion resistance compared to other polymers, hence making it suitable for a wide range of applications [1]. PU can exist in various forms and it can be made to be either rigid or flexible. Due to this, PU can be found in a wide range of applications including elastomers, adhesives, foams, paints, sealants, and others. Conventionally, the raw material for PU, which is polyol, is derived from petrochemical-based products. It is a well-known fact that they are non-biodegradable and non-renewable, and the price fluctuates based on the oil price in the market. With the increasing awareness of environmental issues, recent developments have been focusing on bio-based PU as an alternative to petrochemical-based PU [2]. Bio-based materials possess many advantages such as abundance in nature, making them cheaper, with non-toxic and bio-degradability characteristics, which makes them environmentally friendly [3]. Among all available sources for bio-based PU, vegetable oil is the most attractive choice. Vegetable oil is relatively inexpensive and eco-friendly with the ability to produce PU with good mechanical and physical properties [4].

Recent studies reported some of the vegetable oils used as raw material for polyol synthesis, including soybean oil [5], jatropha oil [6], palm oil [7], canola oil [8], linseed oil [9], and castor oil [10]. The production of polyol extracted from soybean oil was developed in Europe and America a long time ago and used by industries on a large scale to replace petrochemical polyol. In Malaysia, the Malaysian Palm Oil Board (MPOB) and other organizations have intensively researched palm oil-based polyol over 10 years. Currently, palm oil is one of the common vegetable oils used to produce polyol that can be applied as a PU paint resin with two-component systems that are suitable for both interior and exterior applications [11]. Jatropha oil is still new as a raw material for synthesizing polyol in Malaysia and only a few studies have reported on the use of jatropha oil for PU production for different applications so far, mainly studied by our group [12,13,14,15,16]. The usage of non-edible jatropha oil is a new alternative to reduce the dependency on edible oil for polymer production. Unlike palm oil, soybean oil, and corn oil, jatropha oil gives advantages as this can avoid competition to food consumption. With the high degree of unsaturated fatty acids, the double bond in the oil triglyceride structure could be functionalized to the hydroxyl group in polyol preparation which is the initial step of producing PU.

One interesting application of PU is in the preparation of polymer electrolytes or specifically solid polymer electrolyte (SPE). SPE is a solid-state electrolyte made up of salt(s) dispersed into a polymer matrix. SPE became the focus of research studies due to its huge potential in replacing liquid electrolytes in electrochemical devices [17]. SPEs can improve the weaknesses of liquid electrolytes such as leakage, reaction with electrodes, and flammability issues [18]. As a result of these findings, many modifications have been made by different research groups with the aim of improving the properties of the polymer electrolytes such as mechanical, electrochemical, and thermal stability. To achieve this, the amorphous nature of the polymer and low transition temperature (*T_g_*) are considered vital properties as this state can increase the motion of the polymer chain hence increasing the mobility of the ions. Various types of polymers have been investigated and used as the matrix for this purpose such as poly(ethylene oxide) (PEO), poly(acrylonitrile) (PAN), poly(methylmetacrylate) (PMMA), poly(vinylidene fluoride) (PVF), and many more. In essence, PU is a good candidate to be the host polymer of the polymer electrolytes because of their good chemical stability, excellent mechanical properties, and low glass transition temperature *(T_g_*), as well as having a unique multiphase structure consisting of hard and soft segments [19]. Polymeric solvent properties of the soft segment of PU solvates the cations while the hard segments maintain the electrochemical stability [20]. On a side note, lithium salts are the most frequently used dopant in the preparation of SPE. Examples of these salts are: lithium bis(trifluoromethane sulfonyl) imide (LiTFSI) [21], lithium hexafluorophosphate (LiPF_6_) [22], lithium perchlorate (LiClO_4_) [23], and lithium iodide (LiI) [24]. The structure of the anion greatly affects the solubility of salts in the polymer electrolyte system. Salts with large anions, such as ClO_4_^−^ and TFSI^−^, exhibited better solubility than ones with small anions, such as Cl^−^ [25]. However, this depends on the suitability and compatibility of the host polymer and the salts used. The polymer electrolytes are prepared either by a solvent-free, in-situ process, or the conventional solution casting method.

In this study, bio-based polyurethane was used as the polymer host for SPE. The polyol for polyurethane production was prepared from jatropha oil by epoxidation and hydroxylation reaction. Further, the polyol was reacted with the isocyanate group. The polyurethane electrolyte was prepared by solution casting methods with the addition of LiClO_4_ salts at different weight percentages. The samples were characterized by NMR, FTIR, DSC, DMA, and electrochemical analysis, to confirm their chemical structure and properties [26]. The main functional groups for every step of the reaction of polyurethane preparation was observed through NMR analysis to confirm whether the desired compounds are successfully synthesized, while DSC and DMA were used for analysis of thermal properties of the samples including the *T_g_* value and the decomposition temperature. Lastly, ionic conductivities of the samples were tested using alternating current and ionic conductivity was expected to be high, requiring at least ~10^−5^ Scm^−1^ for it to be suitable for practical use.

## 2. Materials and Methods

### 2.1. Materials

Jatropha oil (acid value of 10.5 mg KOH/g, iodine value of 97.1–111.6 g I_2_/100 g) was supplied by Biofuel Bionas Sdn Bhd, Kuala Lumpur, Malaysia. Hydrogen peroxide (30%), formic acid (98%), diphenylmethane 4, 4′ diisocyanate (MDI) and tolylene-2,4-diisocyanate (TDI) were purchased from Merck, Germany. Lithium perchlorate (LiClO_4_) and ethylene carbonate (EC) were obtained from Sigma-Aldrich (St. Louis, MO, USA). Acetone (98%) was supplied by SYSTERM ChemAR (Kielce, Poland). All chemicals and reagents were used as received.

### 2.2. Synthesis of Polyurethane Polymer Electrolytes Matrix

Jatropha oil-based polyol was synthesized in bulk according to procedures that have been previously described in the literature [27,28] with slight modification. In this study, the epoxidation reaction was carried out using a molar ratio of 1.0:0.6:1.7 for oil double bonds to formic acid and hydrogen peroxide. In the preparation of PU, two isocyanates were used which were MDI and TDI. The ratio of polyol to isocyanate was varied to find the best formulation for producing a good film. This film preparation was done by using the solution casting method. Upon obtaining the best ratio, the PU electrolyte was prepared by mixing the polyol with the isocyanate with the addition of acetone as a solvent. The reaction mixture was stirred continuously in an inert condition with the flow of nitrogen gas continuously for one hour in room temperature condition. As the formation of urethane linkage was confirmed, the lithium perchlorate-ethylene carbonate (LiClO_4_–EC) mixture was added to the system and the reaction was continued for another hour. Beforehand, the LiClO_4_–EC mixture was prepared in acetone. The amount of LiClO_4_ salt used was varied from 10% to 30 wt.% while the percentage of EC was fixed to 20 wt.% of the sample. After the reaction was completed, the polymer electrolytes were cast in a teflon mold and left to evaporate in a desiccator for 24 h. Then, the thin film was used for characterization.

### 2.3. Characterizations

Nuclear magnetic resonance (NMR) spectroscopy is a very important characterization to confirm whether the structure of PU has been successfully synthesized. The sample weight of 2.5 mg was diluted in deuterated chloroform (CDCl_3_) solvent. NMR analysis was performed using a JOEL FT NMR spectrometer with 500 MHz frequency. FTIR analysis was performed using a Perkin Elmer 1000 Series equipped with an attenuated total reflection (ATR) accessory in the range of 4000 cm^−1^ to 400 cm^−1^ with 4 cm^−1^ spectral resolution to observe the interaction that occurs in the polymer electrolyte system.

A differential scanning calorimeter (DSC), Mettler Toledo model DSC 822, was used to determine the thermal stability of the samples and is also vital for identification of phase transformation. The heat flow, glass transition temperature (*T_g_*), and the melting point (*T_m_*) in the function of temperature can be determined by using this method. The sample was analyzed at a temperature range of −50 °C to 140 °C at a 5 °C min^−1^ scanning rate. DMA analysis was carried out by using DMA Q800 V20.24 (TA Instruments). A rectangular specimen of 10 mm × 5 mm × 0.5 mm (length × width × height) was analyzed under tension mode with the configuration at 1 Hz, a heating rate of 5 °C min^−1^, with the temperature range of −30 to 140 °C. The storage modulus (E’), loss modulus (E”), and loss factor (tan δ) of the PU and polymer electrolyte films were measured as a function of temperature.

The ionic conductivities of the PU electrolyte matrix were measured by AC impedance spectroscopy on electrochemical measurement. A HIOKI 3532 LCR HiTESTER was set from 50 Hz to 1 MHz at 1000 mV amplitude and the test was carried out at room temperature. The sample was punched into a round shape of 16 mm in diameter and then was sandwiched between two stainless steel (SS) block electrodes. The bulk resistance (*R_b_*) was measured and then used to calculate the ionic conductivity (*σ*) with the following formula:

(1) $$\sigma =\frac{l}{A\times R_{b}}$$

where *l* is the thickness of the sample (cm), and *A* is the contact surface area (5.13 cm^2^). From the impedance data, the dielectric study of the PU electrolytes was studied in terms of dielectric constant, dielectric loss, and tan delta. The temperature dependence of the highest conducting sample was tested from room temperature to 100 °C with an applied frequency of 50 Hz to 1 MHz at 1000 mV amplitude.

The swelling behavior of the polymer electrolyte was determined using ASTM D570 for the water absorption test. The PU film was weighed and then immersed in distilled water for 24 h in room temperature conditions. It was then dried by using filter paper and the mass was weighed again. The procedure was triplicated over a duration of eleven days, and the mass change was recorded. The degree of swelling (*DS*) is calculated as follows:

(2) $$DS=\frac{W_{w}-W_{d}}{Wd}\times 100$$

where *W_w_* represents the initial weight and *W_d_* is the weight after the immersion procedure.

## 3. Results and Discussion

### 3.1. NMR Analysis of EJO, Polyol, and PU

#### 3.1.1. ^13^C NMR of Jatropha Oil, Epoxidized Jatropha Oil, and Polyol

**Figure 1:**
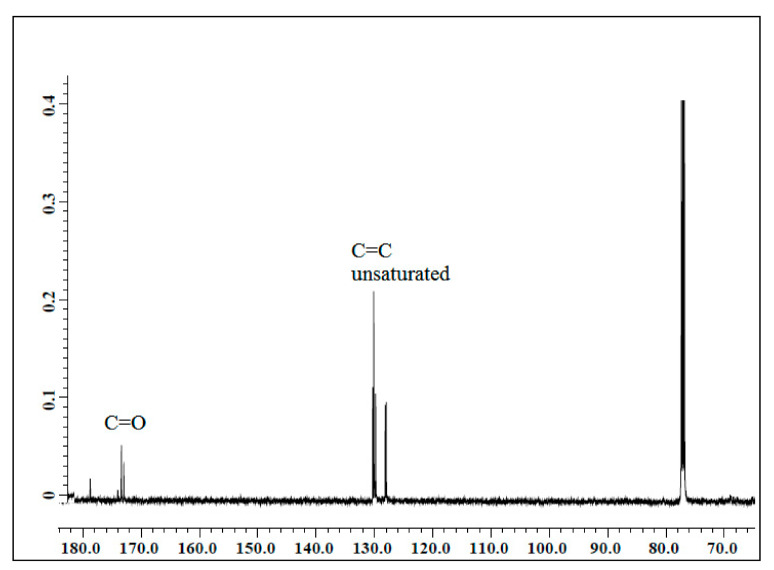
^13^C NMR spectrum of jatropha oil.

**Figure 2:**
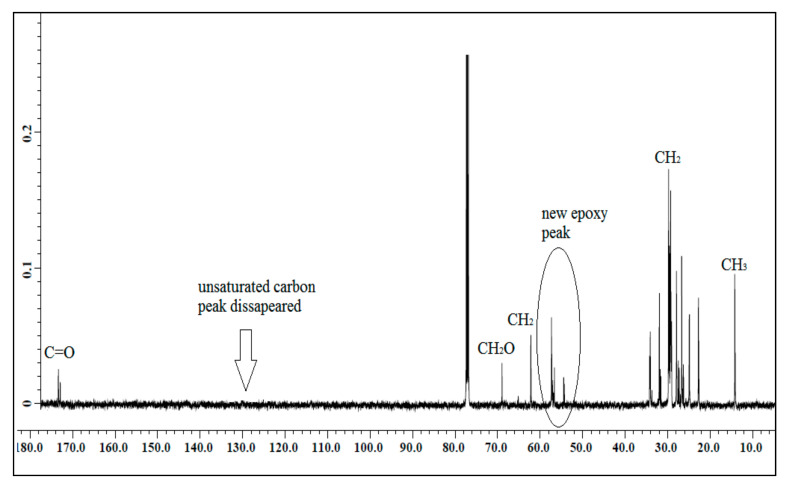
^13^C NMR spectrum of epoxidized jatropha oil.

**Figure 3:**
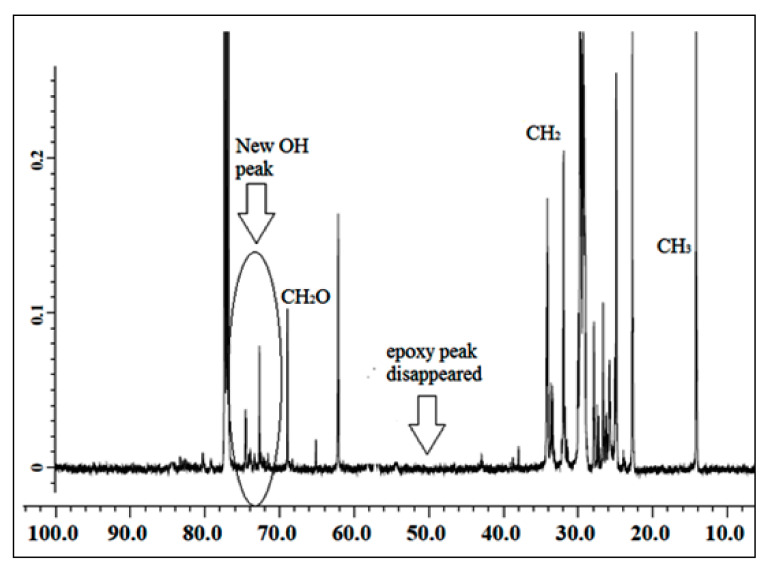
^13^C NMR spectrum of polyol.

The NMR spectroscopic characterization of jatropha oil, epoxidized jatropha oil, and polyol was carried out to confirm whether the desired product was successfully synthesized. Figure 1 presents the ^13^C NMR spectrum of jatropha oil. Upon the completion of the epoxidation reaction, the formation of the epoxy ring was confirmed as shown in Figure 2. From the spectrum, the new signal was formed, indicating carbons of the epoxy ring, verified at 56.3–57.0 ppm, while the signal for unsaturated fatty acids at 128 ppm was observed to have disappeared. In this case, it can be confirmed that the double bond of jatropha oil converted to an epoxy ring after the epoxidation reaction took place. Based on the figure, the peak observed at 15 ppm was assigned for terminal carbon of methyl groups (CH_3_) and signals varying from 22.79–29.78 ppm were related to methylene carbons of the long carbon chain of the epoxidized oil. Meanwhile, the signals observed at 174.2 and 62.1 ppm indicated the ether and ester carbonyl. Those peaks show no changes before and after the epoxidation reaction. The polyol ^13^C NMR is shown in Figure 3. Based on the figure, the characteristic jatropha oil-based polyol had a new signal in a range of 75–78 ppm indicating carbon attached to hydroxyl groups. On the other hand, the signal at 56.3–57.0 ppm which was assigned to the epoxy carbon was found to be absent.

#### 3.1.2. ^1^H NMR of Jatropha Oil, Epoxidized Jatropha Oil, and Polyol

**Figure 4:**
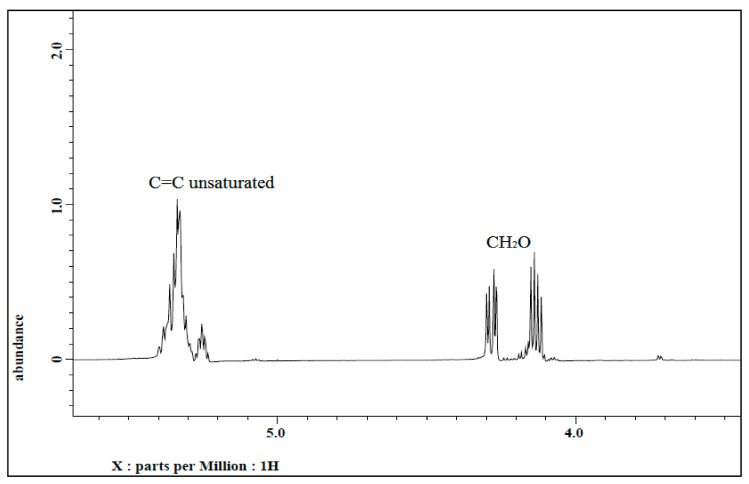
^1^H NMR spectrum of jatropha oil.

**Figure 5:**
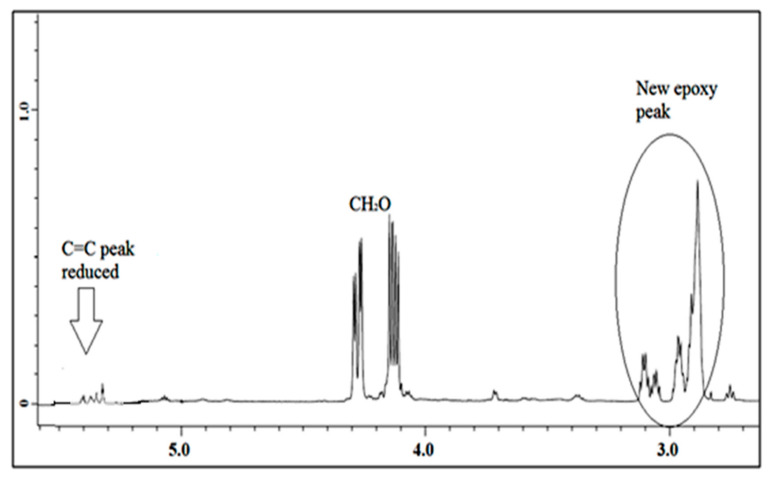
^1^H NMR of epoxidized jatropha oil.

^1^H NMR spectra of jatropha oil and epoxidized jatropha oil are depicted in Figure 4 and Figure 5. Based on the figures, the new signals indicated that protons of epoxy rings appeared at 2.9 ppm. Due to the epoxidation reaction, the characteristic peak of the unsaturated hydrogen atoms which present at 5.7–5.8 ppm was found to have disappeared and these findings suggested that a complete conversion of the carbon-carbon double bonds had taken place. The signals between 4.0–4.3 ppm corresponded to the characteristic of hydrogen of CH_2_ and CH glycerol groups of the triglycerides structure. In addition, a triplet of α-CH_2_ protons observed at 2.0–2.8 ppm showed the presence of long-chain methyl esters in epoxidized jatropha oil structure. Other significant peaks were observed at 0.84 ppm which is assigned to terminal methyl protons, and also a strong signal at 1.2 ppm related to methylene protons of the carbon chain. These signals showed no changes before and after the epoxidation reaction took place. A similar ^1^H NMR spectrum of epoxidized jatropha oil was also reported previously [29,30].

**Figure 6:**
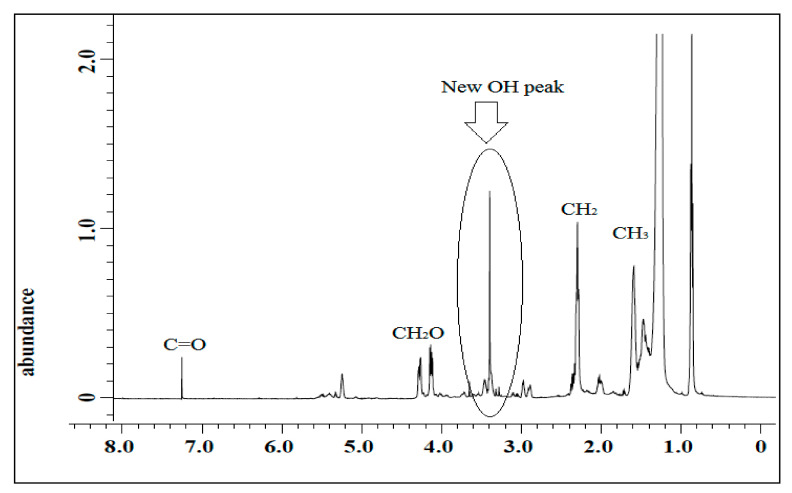
^1^H NMR spectrum of jatropha oil-polyol.

The spectrum of jatropha oil-based polyol is shown in Figure 6. Based on the figure, a single sharp signal at 3.60 ppm was observed and it was assigned to the hydrogen of hydroxyl (CH_2_OH) compound. The characteristic peak at 2.90 ppm for epoxy hydrogen was observed to show a reduction in its intensities and almost disappeared. These findings prove that jatropha oil-based polyol was successfully synthesized.

#### 3.1.3. ^1^H NMR of PU and PU-25LiClO_4_

**Figure 7:**
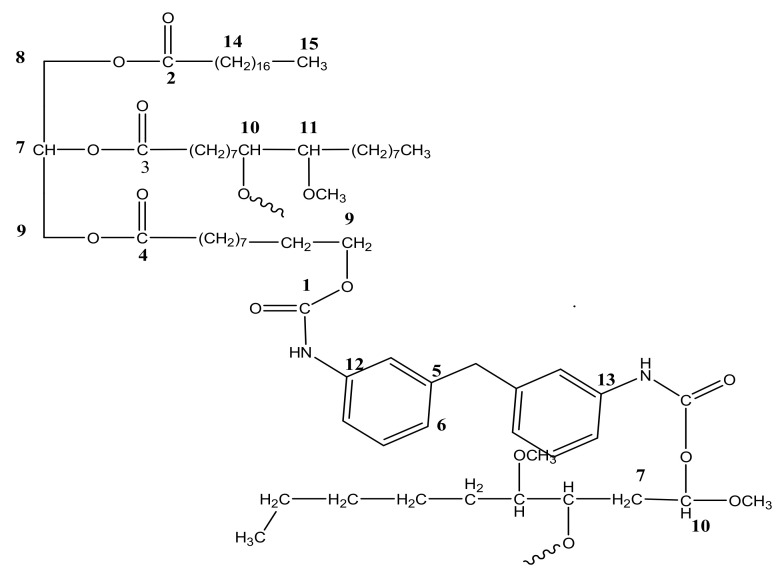
Chemical structure of polyurethane (PU) based on NMR spectra.

**Figure 8:**
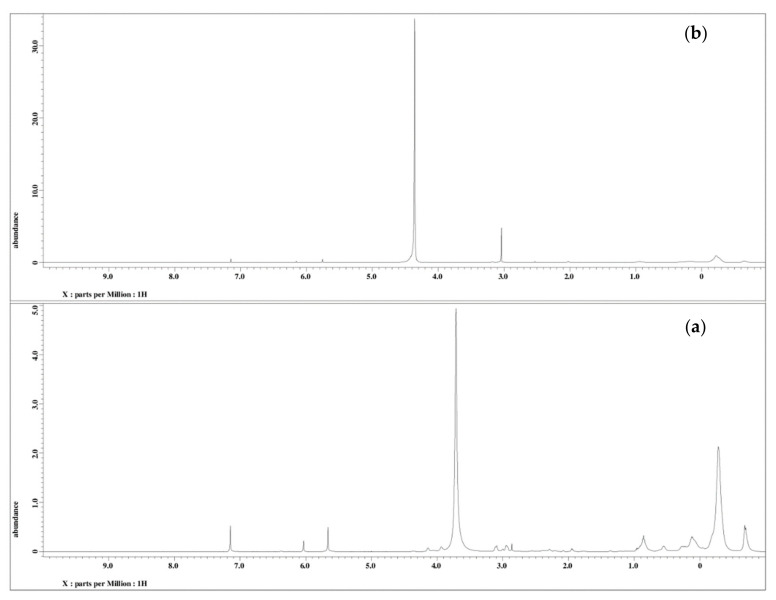
^1^H NMR spectra (**a**) PU and (**b**) PU-25LiClO_4_.

The ^1^H NMR analysis of PU and PU-25LiClO_4_ was used to confirm and prove the formation of polymer-salt complexes. The proposed structure of PU is presented in Figure 7. Based on the figure, the formation of urethane linkages is suggested in between carbon 9 and 10 which is located at the OH positions of polyol in the middle of the chains. A similar pattern of NMR spectra was also reported in the literature [7] on PU synthesized from palm kernel oil. There were some changes in the NMR spectrum of the PU host polymer after the addition of salts as shown in Figure 8. Based on the figure, upon the addition of 25 wt.% LiClO_4_ into the PU, the proton peaks that contain nitrogen and oxygen atoms, located at 7.0–8.0 (HNCO), 5.6–5.7 (CH_2_CH_2_N), 2.9–3.0 (CH_2_O), and 1.5–1.6 (CH_2_CH_2_O) were observed. Moreover, the intensity of these characteristic peaks was observed to decrease after the addition of LiClO_4_ into the host polymer. This was believed to happen due to the interaction of salts with the PU host polymer to form polymer-salt complexes. It happened because oxygen and nitrogen atoms in the PU structure act as electron donors to the Li^+^ ions. On the other hand, there was a new peak observed at 3 ppm in the polymer electrolyte which might be because of the increase in the number of lithium ion interactions with the EC plasticizer. A similar observation was also reported previously [31] in work on gel polymer electrolyte using LiClO_4_ salt.

### 3.2. Observation of PU with Different NCO/OH Ratios

In the preparation of PU, the optimal ratio of isocyanate to polyol (NCO/OH) must be determined to make sure the electrolyte film produced has good characteristics such as being flexible, stable, not sticky, and easy to release from the mold. Table 1 shows the observation of the polyol with the isocyanate ratio for production of PU solid polymer. Based on the table, aromatic isocyanates MDI and TDI were chosen as components for the hard segment in the PU elastomer as both chemicals were reported to give better mechanical strength to the PU as compared to aliphatic isocyanates such as isophorone diisocyanate (IPDI) or 1,6-hexamethylene diisocyanate (HDI) [32].

Based on the observation, the best PU was obtained from PU/MDI3 and PU/TD12 and was flexible, not sticky, and easy to release from the mold. However, from the two, PU/MDI3 was chosen to be the PU host polymer for several reasons. Firstly, MDI-based PU is less toxic than TDI which has a high vapor pressure. The lesser toxicity in MDI-based PU is very important for environmental and health concerns. Furthermore, the chemical reactions between MDI and polyol to synthesize PU can happen at a low reaction temperature of around 40–65 °C compared to TDI which only reacts at higher temperatures typically from 60 °C to 75 °C [32]. Thus, the PU/MDI3 was selected to be the host for the polymer electrolyte film. The polymer electrolyte was prepared by doping 20 wt.% of EC and 10 wt.% to 30 wt.% of LiClO_4_.

### 3.3. Water Absorption Test

**Figure 9:**
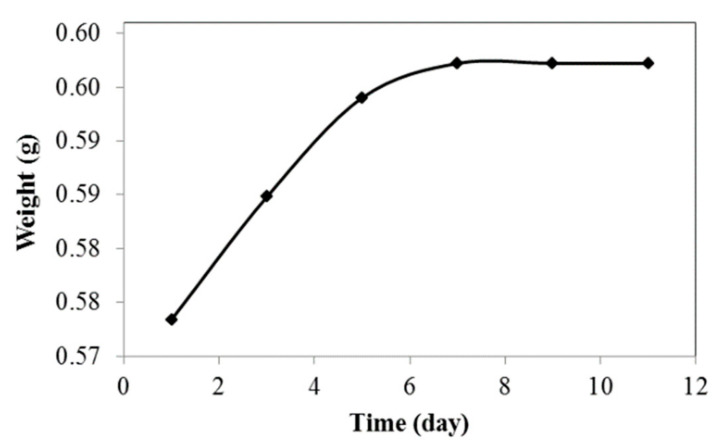
Water absorption of PU.

Water absorption properties of the jatropha oil-based PU were investigated to determine the amount of water absorbed at room temperature over several days. As shown in Figure 9, the sample could absorb a small amount of water which was in the range of 4.15% within 9 days. The sample weight was constant after that, indicating that jatropha oil-based PU has low water absorption and is hydrophobic in nature. The long aliphatic chain and less polar molecules make PU a hydrophobic polymer. Its high water barrier properties make it suitable for application in insulation materials, coatings, and electrochemical devices [33].

### 3.4. FTIR Analysis

**Figure 10:**
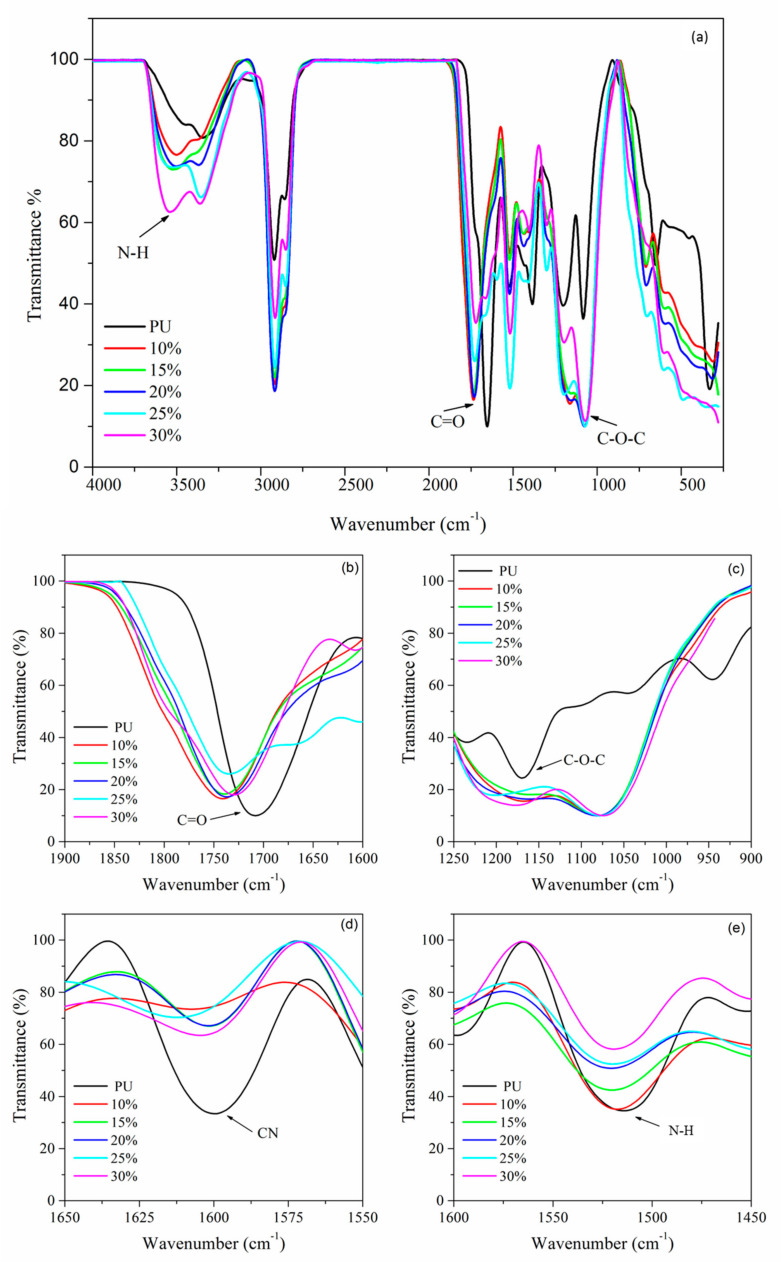
FTIR spectra of PU, PU electrolytes with 10 wt.% to 30 wt.% LiClO_4_ at (**a**) full view, (**b**) C=O, (**c**) C‒O‒C, (**d**) CN and (**e**) N‒H region.

The FTIR analysis was done to investigate the effect of the salt on the PU structure to prove the formation of polymer-electrolytes complexes. Figure 10 depicts the FTIR spectra of PU and PU-LiClO_4_ at different LiClO_4_ contents. The functional groups of carbonyl (C=O) (1750–1650 cm^−1^), ether and ester group (C–O–C) (1300–1000 cm^−1^), amine group (N–H), and cyanide group (CN) (1550–1500 cm^−1^) are being focused on as they correspond to oxygen and nitrogen atoms in the PU molecule. According to the literature, the oxygen and nitrogen atoms in the PU molecule are responsible for coordinating with lithium ions from the doping salt to form polymer electrolytes complexes [34,35].

The C=O group region of PU showed shifting to the lower wavenumber upon the addition of salt, shifting from 1740.92 cm^−1^ to 1726.74 cm^−1^ for PU and 25 wt.% LiClO_4_, respectively. The non-hydrogen-bonded C=O also shows decreasing intensities. For instance, the intensities of the C=O absorption band of 25 wt.% LiClO_4_ were seen to be lower compared to PU, as shown in Figure 10b. These changes indicated that the C=O region was highly affected by the introduction of LiClO_4_ to the PU host polymer, possibly because of the coordination of Li^+^ with carbonyl oxygen atoms [35]. On the other hand, the absorption peak at 1076.00 cm^−1^ which corresponded to the C–O–C group of PU shifted to 1071.64 cm^−1^ with the addition of 25 wt.% LiClO_4_. Figure 10c shows the significant shifting of the C–O–C group vibration band after the addition of the salt to the PU host polymer. Both shifts of C=O and (C–O–C) regions indicated that there were strong intermolecular interactions between lithium ions and oxygen atoms in the PU host polymer. The oxygen atoms in C=O and C-O-C functional groups in PU structure were suggested to act as electron donors to the LiClO_4_ doping salt and hence formed coordination bonds to form polymer electrolyte complexes. It was also noted that the band at 1607.36 cm^−1^ which was assigned to the CN functional group in PU also showed shifting upon the addition of LiClO_4_ salt as shown in Table 2. It is also important to note that the CN vibration peak of polymer electrolyte became almost a plateau upon the increases of the concentration of lithium ion shown in Figure 10d. The same observation was seen for the NH group at 1650–1500 cm^−1^ regions. The shifting to the lower wavenumber was observed from 1528.93 cm^−1^ to 1520 cm^−1^ for PU and 30 wt.% LiClO_4_-PU, respectively. The intensities of the NH band were seen to decrease upon the addition of the doping salt as illustrated in Figure 10e. With these observations, it is worth mentioning that nitrogen atoms from CN and NH were acting as electron donors to form a coordinate bond with lithium doping salt to form polymer electrolyte complexes.

Next, there were also exhibit changes in non-hydrogen bonded NH at region 3400–3200 cm^−1^. The N–H peak of PU at 3300 cm^−1^ shifted to a higher wavenumber with the addition of the salts. It was also observed that the peak became broad as the concentration of the LiClO_4_ increased. This could be explained based on the interaction of Li^+^ and Na^+^ ions with the N atoms of free –NH groups. The N–H bond becomes weak as a result of the interaction of cations with the lone pair of electrons on the nitrogen atom. The findings were similar to the effect of LiClO_4_ ion on the PU polymer host as reported previously [36]. It could also be due to interaction of the cations with the non-bonded electrons of the carbonyl oxygen atoms that leads to reducing the H-bond strength. From these FTIR results, it can be concluded that the interactions between the host polymer and salts occurred in both the hard segment (C=O and N–H) and soft segment (C–O–C) of PU. The overall IR spectra show a similar pattern after the addition of salt into the polymer host as seen in previous research [35,36].

### 3.5. EIS Analysis

Table 3 shows the results of the ionic conductivity obtained from the impedance data [26]. The highest conductivity at room temperature of PU-LiClO_4_ is achieved at 25 wt.% of LiClO_4_ doped in polyurethane which is 1.29 × 10^−4^ Scm^−1^. The ionic conductivity increases from the blank polyurethane until 25 wt.% of LiClO_4_. The increment could be explained by the increase of mobile charge carriers generated by the dissociation of LiClO_4_ salt. Beyond the optimum value, an increase in the amount of salts could lead to the presence of excess mobile charge carriers. They could form ion pairs, which in turn would disturb the movement of the mobile charge carriers, thus reducing the ionic conductivity [37].

### 3.6. Temperature Dependence Analysis

**Figure 11:**
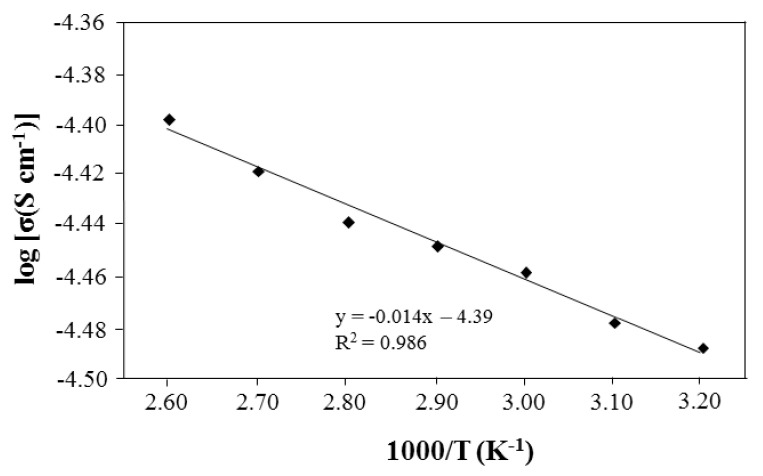
The Arrhenius plot of PU-25LiClO_4_.

**Figure 12:**
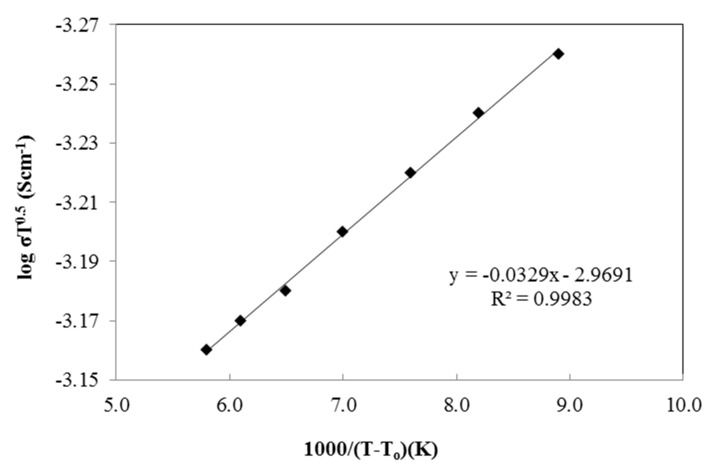
The VTF plot of PU-25LiClO_4_.

The temperature dependence test was performed from 300 K to 373 K to study the effect of temperature on the ionic conductivity of the polymer salt complexes. The test was done using 25 wt.% of PU-LiClO_4_ sample which has the highest ionic conductivity at room temperature. The temperature dependence of the ionic conductivity is generally shown by either an Arrhenius equation or a Vogel–Tamman–Fulcher (VTF) equation. The Arrhenius graph in Figure 11 gives an imperfect straight-line graph with a regression line of 0.986 and shows non-linear behavior. The VTF model was then suggested and the graph is shown in Figure 12. Based on the figure, the regression line was observed to be close to unity, at 0.998. The PU-LiClO_4_ polymer electrolyte was suggested as obeying the VTF model by temperature dependency and hence suggesting the presence of an amorphous structure in the solid polymer electrolyte. The VTF equation was given by:

(3) $$\sigma =A\left(\sqrt{T}\right)\exp\;[-E/(T-T_{o})]$$

where *A* is a constant, *E* is the activation energy, and *T_o_* is the equilibrium state of the glass transition temperature. The conductivity was found to rise with the temperature, due to polymer expansion, and produced free volumes in the polymer chains. This free volume gave mobility to the ions to migrate and move, hence increasing the ionic conductivity. This phenomenon supports the DSC analysis which mentioned that the glass transition of the polymer electrolyte keeps decreasing along with the increasing of ionic conductivity. The decreases in *T_g_* value are also caused by the movement of the polymer chains which gave free volumes. The polymer chains will obtain high internal mode, making the rotation chain faster. Therefore, the movement of chain segments will increase and thus increase the ion conductivity of the polymer electrolyte. A similar pattern of behavior of ionic conductivity towards temperature which obeys Arrhenius law was also reported by [38].

### 3.7. Dielectric Properties

**Figure 13:**
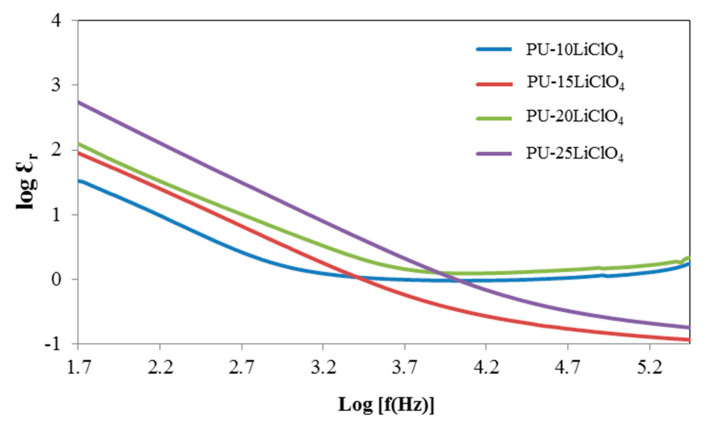
Dielectric constant against log frequency of PU having 10 wt.% to 25 wt.% LiClO_4_.

**Figure 14:**
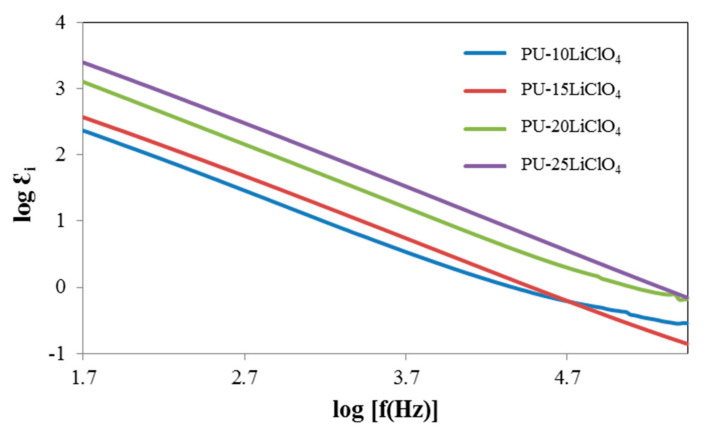
Dielectric loss against log frequency of PU having 10 wt.% to 25 wt.% LiClO_4_.

The dielectric constant (*Ɛ_r_*) is a measurement of stored charge in the material. The graphs of dielectric constant (*Ɛ_r_*) and dielectric loss (*Ɛ_i_*) versus log frequency were plotted and are shown in Figure 13 and Figure 14. The equation used to calculate the *Ɛ_r_* and *Ɛ_i_* is shown as follows:

(4) $$\varepsilon_{r}=\frac{Z_{i}}{\omega C_{o}\left(Z_{r}^{2}+Z_{i}^{2}\right)}$$

(5) $$\varepsilon_{i}=\frac{Z_{r}}{\omega C_{o}\left(Z_{r}^{2}+Z_{i}^{2}\right)}$$

where *C_o_* = *Ɛ_o_A*/*t*, *Ɛ_o_* = permittivity of free space, and ω = 2π*f*.

Based on Figure 13 and Figure 14, the values of log *Ɛ_r_* and log *Ɛ_i_* are high at the low frequency region (1.5 Hz). This trend was observed because of the electrode polarization [39]. While at high frequency, log *Ɛ_r_* and log *Ɛ_i_* started to decrease and the periodic reversal of electric field happened quickly as there was no excess ion diffusion in the direction of the field [39,40]. Furthermore, as the amount of LiClO_4_ salt increased, the value of log *Ɛ_r_* and log *Ɛ_i_* was increased from 10% PU–LiClO_4_ to 25% PU–LiClO_4_. The increasing value of both log *Ɛ_r_* and log *Ɛ_i_* were due to the charge carrier density, *n* [41] and it can enhance the conductivity since it is directly proportional to *n*. Hence, with the increase in the charge carrier density, ionic conductivity will be increased.

Based on the impedance data analysis, dielectric loss tangent (tan δ) was determined by using the following equation:

(6) $$\tan\delta =\frac{Z_{r}}{Z_{i}}$$

**Figure 15:**
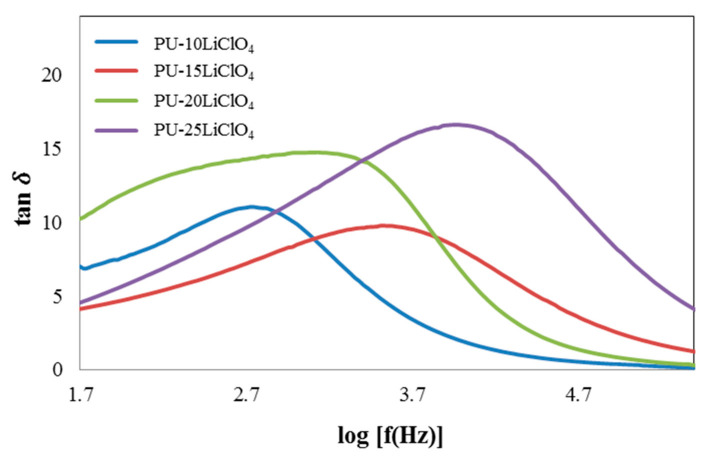
Tan δ versus log frequency of PU having 10 wt.% to 25 wt.% LiClO_4_.

**Figure 16:**
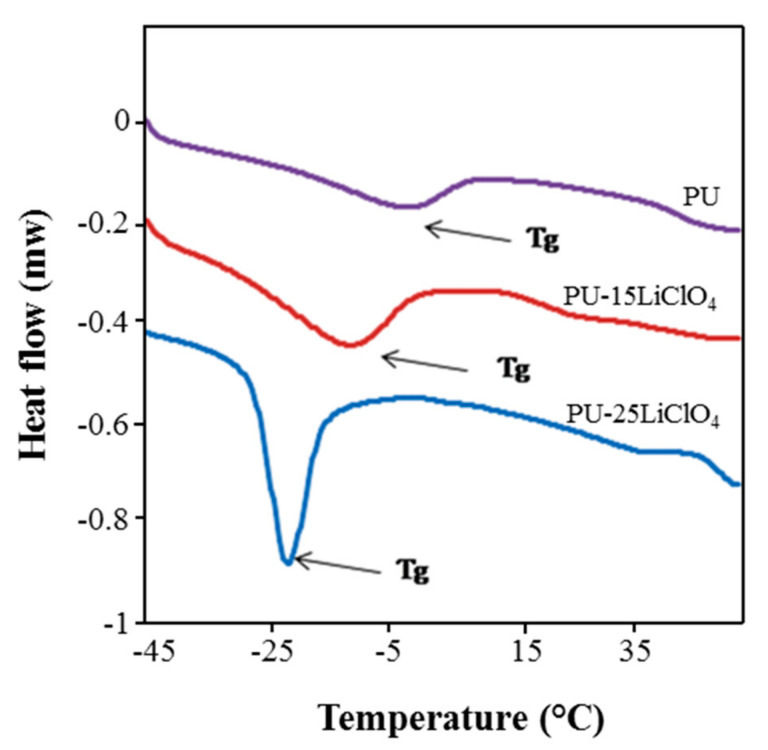
DSC thermograms of PU, PU-15LiClO_4_, and PU-25LiClO_4_.

The relaxation time (τ) can be calculated from the maximum log frequency of the peak for different percentages of LiClO_4_ doped into PU. Figure 15 shows the loss tangent curve of the PU–LiClO_4_ polymer electrolytes and the relaxation parameters are displayed in Table 4. Based on the graph, the peaks shifted toward the right side as the percentage of LiClO_4_ doped into PU increased from 10% up to 25%. The relaxation time taken decreased [40] as the peaks shifted toward the right. Figure 16 shows that 25 wt.% PU–LiClO_4_ had the lowest relaxation time which was 1.03 × 10^−4^ s which is in line with the conductivity measurement data that were obtained at 1.29 × 10^−4^ S cm^−1^.

### 3.8. DSC Analysis

DSC analysis was done to analyze the thermal behavior of the PU and polymer salt complexes. The changes in *T_g_* are presented in Figure 16 and the data are provided in Table 5. The *T_g_* of jatropha oil-based PU was observed at −5 °C, while the melting temperature (*T_m_*) was recorded at 196 °C. The low *T_g_* below room temperature was suggested as resulting from the dominant flexible segment which comprised of the polyol backbone in the PU structure. The addition of salts into the PU was proven to affect the *T_g_* and *T_m_* values of the polymer salt complexes as shown in the Figure 16. It can be seen that the *T_g_* of PU decreased to −22.06 °C after the addition of 25 wt.% of LiClO_4_. Not only that, but the *T_m_* value was also seen to decrease upon the addition of salts as mentioned in the Table 5. The shifting in *T_g_* and *T_m_* values to lower temperature indicated that the polymer salts segment became less rigid in amorphous phase structure, which has been supported in XRD analysis [28], hence increasing the segmental mobility of PU salt complexes and then decreasing the *T_g_* value. Moreover, the decrease in *T_g_* and *T_m_* of the polymer host was also related to the increasing of ionic conductivity for the polymer electrolytes. The incorporation of the salt into PU causes the weakening of the dipole–dipole interactions between the PU chains, and hence makes the ions move freely through the polymer chain network when an electric field is applied and hence increases the conductivity [35]. A similar trend of results which showed decreasing in *T_g_* and *T_m_* value for bio-based PU after the addition of the salts was reported by [34,35].

### 3.9. Dynamic Mechanical Analysis (DMA)

**Figure 17:**
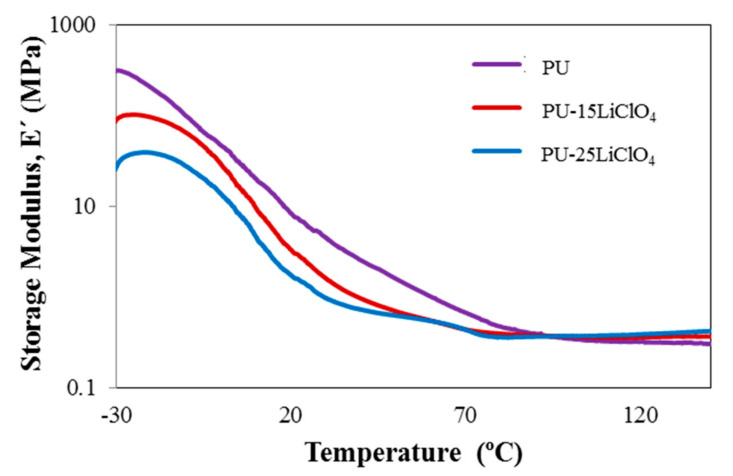
Storage modulus of PU, PU-15LiClO_4_, and PU-25LiClO_4_ as a function of temperature.

**Figure 18:**
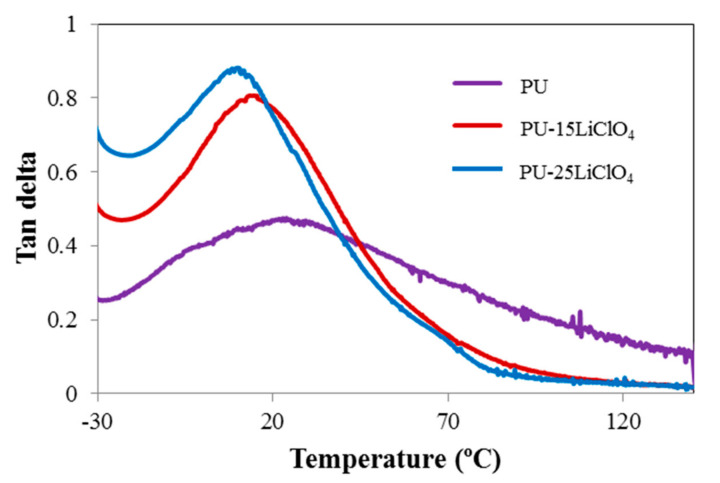
Tan delta of PU, PU-15LiClO_4_, and PU-25LiClO_4_ as a function of temperature.

The dynamic mechanical behavior of the PU and electrolyte films was investigated by a dynamic mechanical analyzer. Figure 17 and Figure 18 show the storage modulus and tangent delta as functions of temperature for the PU and PU–LiClO_4_ films, respectively. The films are in a glassy state at a temperature below −30 °C. As the temperature increased the storage modulus (*E*’) was observed to be significantly decreased and flattened as the materials become more loose when heated; hence the capacity for energy absorption was diminished [42]. The drop of the *E*’ upon the increase of temperature was also attributed to the glass-rubber transition as tan δ reaches its maximum peak simultaneously as shown in Figure 18.

The magnitude of E’ in PU was observed to be higher than in PU-LiClO_4_ polymer electrolyte, indicating that the sample was in higher dynamic stiffness and had strong hydrogen bonding in the polymer chain compared to polymer salt complexes. The *T_g_* values obtained from DMA and DSC are presented in Table 6. The difference between the *T_g_* values obtained from DSC and DMA was caused by the choice of different frequencies employed in both measurements and due to the different nature of both measurements. The *T_g_* in DMA measurement was identified from the peak maximum (α relaxation) of tan δ as many studies have reported that the tan δ peak (damping peak) was associated with the glass *T_g_.* Furthermore, in this study, the tan δ peak was observed compared to storage modulus and loss modulus peaks.

Based on the table, *T_g_* values for both DSC and DMA measurement were seen to be decreased upon the addition of the LiClO_4_ ion. The shift of *T*_g_ value of PU towards a lower temperature indicated that the chain mobility of PU was increased upon the addition of salts, and the segment became less rigid. It is also suggested that it is caused by the plasticizing effect from the addition of EC [43]. The low *T_g_* after the addition of the salt into the PU was also related to the higher ionic conductivity in polymer salt complexes compared to the PU host polymer. This was due to the incorporation of the salt into the PU polymer host resulting in the weakening of the dipole–dipole interactions between the PU chains, making the ions move freely through the polymer chain network when an electric field is applied, and then increasing the conductivity [36].

## 4. Conclusions

Polyurethane from jatropha oil was successfully synthesized and the solid polymer electrolyte was prepared. The properties of the electrolyte were determined by vibrational, thermal, and electrochemical analysis. The FTIR analysis proved the complexation between the salt and PU at the amide, carbonyl, and ether regions. The impedance analysis reveals that the ionic conductivity is dependent on the concentration of the salt. There was an increasing trend of conductivity as the salt content increased and the highest conductivity was achieved at 25 wt.% of LiClO_4_, while beyond that the conductivity decreased. The observation can be related to the dielectric properties of the PU electrolyte. The lowest relaxation time was recorded for the highest conducting sample. This finding can be further supported by the DSC and DMA analysis. The *T_g_* of the electrolyte decreased with increasing salt content simultaneously enhancing the ionic conductivity. Overall, the PU electrolyte shows promising potential for further study in electrochemical device applications.

## Figures and Tables

**Table 1:** NCO/OH weight ratio for PU and its characteristics.

Sample	NCO/OH	Observations
PU/MDI1	1:2.5	Not flexible, too rigid, difficult to remove from the mold
PU/MDI2	1:3.5	Not flexible, rigid, difficult to remove from the mold
PU/MDI3	1:4.5	Flexible, stable, not sticky, easy to remove from the mold
PU/MDI4	1:6.5	Soft, easy to remove from the mold
PU/MDI5	1:8.5	Too soft, a bit sticky, difficult to remove from the mold
PU/TDI1	1:6.0	Not flexible, too rigid, difficult to remove from the mold
PU/TDI2	1:12.0	Flexible, stable, not sticky, easy to remove from the mold
PU/TDI3	1:15.0	Soft, easy to remove from the mold

**Table 2:** FTIR absorption peak of PU and PU/LiClO_4_.

Functional Groups		Wavenumber (cm^−1^)				
	PU	10 wt.%	15 wt.%	20 wt.%	25 wt.%	30 wt.%
C=O	1740.92	1739.97	1731.63	1729.32	1726.74	1727.00
C‒N	1607.36	1604.50	1601.77	1601.56	1600.00	-
N‒H	1528.93	1526.62	1524.75	1523.55	1520.27	1520.00
C‒O‒C	1076.00	1073.08	1072.36	1071.64	1071.33	1070.80

**Table 3:** Ionic conductivity of various wt.% of LiClO_4_ in PU polymer electrolytes.

Sample	Conductivity, σ (Scm^−1^)
PU	3.00 × 10^−9^
PU-10LiClO_4_	2.23 × 10^−8^
PU-15LiClO_4_	2.90 × 10^−7^
PU-20LiClO_4_	2.30 × 10^−6^
PU-25LiClO_4_	1.29 × 10^−4^
PU-30LiClO_4_	2.92 × 10^−7^

**Table 4:** Relaxation parameter of PU having 10 wt.% to 25 wt.% LiClO_4_.

Sample	τ (×10^−4^) s
PU–10LiClO_4_	14.00
PU–15LiClO_4_	2.61
PU–20LiClO_4_	4.17
PU–25LiClO_4_	1.04

**Table 5:** *T_g_* and *T_m_* of PU, PU–15LiClO_4_, and PU–25LiClO_4_.

Sample	*T_g_*	*T_m_* (°C)
PU	−5.0	198.0
PU–15LiClO_4_	−12.7	196.0
PU–25LiClO_4_	−22.1	150.3

**Table 6:** *T_g_* of PU, PU–15LiClO_4_, and PU–25LiClO_4_ recorded by DSC and DMA.

Sample	*T_g_* by DMA (tan δ Peak), °C	*T_g_* by DSC, °C
PU	21.5	−5.0
PU–15LiClO_4_	13.5	−12.7
PU–25LiClO_4_	6.2	−22.1
